# Supplementary material for: “Because I said so.” – Collection and evaluation of parenting phrases in German-speaking samples
Source: PLoS One. 2026 Apr 16;21(4):e0346718. doi: 10.1371/journal.pone.0346718 (PMC13086427; doi:10.1371/journal.pone.0346718)
Supplement: S1 Text — (PDF) [file pone.0346718.s004.pdf]

Correction of the preregistration of Study 1:

Hypothesis 6:

Note that in the preregistration, we stated that for example "high control" phrases would be correlated with the dimension "high control." However, a more correct formulation is that these phrases were expected to be associated with the control dimension, which can manifest at both high and low levels. Accordingly, our hypotheses posit that for example the use of "high control" phrases would be positively associated with higher values on the control dimension, while the use of “low control” phrases is expected to be negatively associated with control. The same applies for the warmth dimension and phrases categorised as high or low in warmth.

Analysis H1 and H2

Please note that in our preregistration, the statistical method for H1 and H2 was specified as independent t-tests. However, because the same participants reported both on the parenting phrases they experienced from their own parents and on the phrases they use with their children, these measures have to be considered dependent and therefore require dependent t-tests.
