# Supplementary material for: “Because I said so.” – Collection and evaluation of parenting phrases in German-speaking samples
Source: PLoS One. 2026 Apr 16;21(4):e0346718. doi: 10.1371/journal.pone.0346718 (PMC13086427; doi:10.1371/journal.pone.0346718)
Supplement: S2 Text — (PDF) [file pone.0346718.s005.pdf]

Study 2: Exploratory analyses of gender differences in parenting phrase ratings

Gender differences concerning the ratings of parenting phrases were analysed using one-sided independent t-tests and a Mann-Whitney U test for control, as the assumption of normal distribution was not met (W = 0.91, p = .030 and W = 0.96, p = .009). Women rated phrases as significantly less warm (t(103) = 2.31, p = .011, 95% CI [0.04, ∞], d = 0.54, , BF = 2.35) and significantly more self-esteem-inhibiting (t(103) = 3.00, p = .002, 95% CI [0.08, ∞], d = 0.73, BF = 14.118) compared to men. No significant difference was found for the control dimension (W = 947, p = .380, 95% CI [-0.12, ∞], , BF = 0.25). Boxplots comparing both groups for all three dimensions can be viewed in Figure S1.

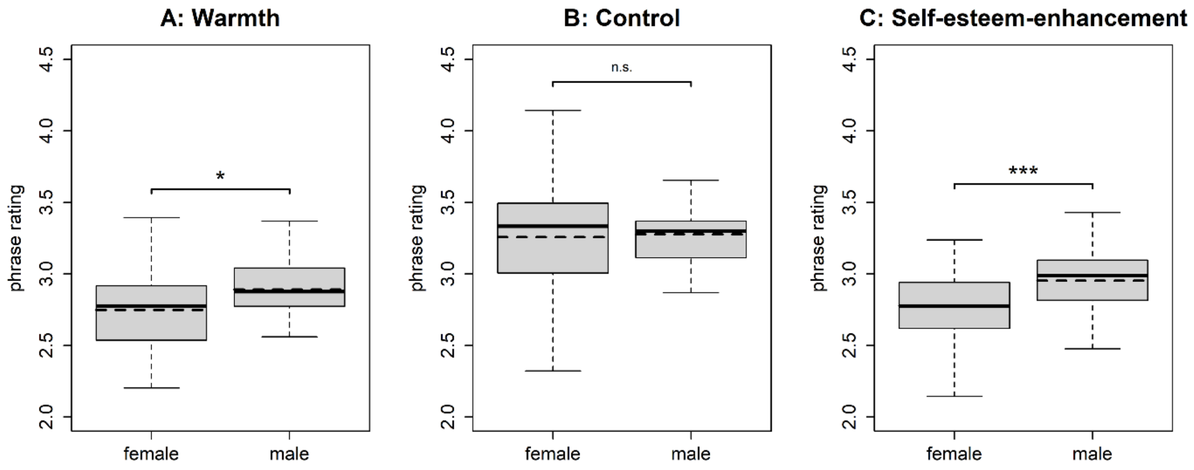

Figure S1.

Boxplots showing gender differences for the ratings on the dimensions warmth, control and self-esteem-enhancement. The dashed line represents the mean, while the solid line indicates the median of the data. Significance levels are denoted as follows: p < .05 (*), p < .001 (**), and n.s. = not significant.
