# Supplementary material for: “Because I said so.” – Collection and evaluation of parenting phrases in German-speaking samples
Source: PLoS One. 2026 Apr 16;21(4):e0346718. doi: 10.1371/journal.pone.0346718 (PMC13086427; doi:10.1371/journal.pone.0346718)
Supplement: S1 Analysis — (PDF) [file pone.0346718.s006.pdf]

Prerequisite analyses Study 1 and results with and without outliers

Hypothesis	Prerequisite analysis	Statistical test used	Number of outliers	Results with outliers excluded	Results all data included
H1	Shapiro-Wilk test (normality of differences):<br>*W* = 0.9638<br>*p* = 0.6223	Dependent t-test	/	/	*t*(19) = 2.041<br>*p* = 0.028<br>95%CI [0.020, inf]<br>mean difference = 0.13<br>*BF* = 1.282<br>*d* = .66
H2	Shapiro-Wilk test (normality of differences):<br>*W* = 0.924<br>*p* = 0.117	Dependent t-test	/	/	*t*(19) = 7.183<br>*p* < .001<br>95%CI [0.346, inf]<br>mean difference = 0.45<br>*BF* = 22022.33<br>*d* = 1.98
H3	Shapiro-Wilk test:<br>Variable 1:<br>*W* = 0.976<br>*p* = 0.874<br>Variable 2:<br>*W* = 0.872<br>*p* = 0.01	Spearman correlation	/	/	*p* = 0.934<br>*ρ* = -0.020<br>95%-CI [-0.458, 0.426]<br>*BF* = 0.476
H4	Shapiro-Wilk test:<br>Variable 1:<br>*W* = 0.88157<br>*p* = 0.01888<br>Variable 2:<br>*W* = 0.943<br>*p* = 0.269	Spearman correlation	/	/	*p* = 0.455<br>*ρ* = 0.177<br>95%-CI [-0.288, 0.575]<br>*BF* = 0.592
H5a	Shapiro-Wilk test:<br>Variable 1:<br>*W* = 0.9699<br>*p* = 0.074<br>Variable 2:<br>*W* = 0.88766<br>*p* < 0.001	Spearman correlation	1	*p* = 0.707<br>*ρ* =-0.04<br>95%-CI [-.272, 0.187]<br>*BF* = 0.285	*p* = 0.567<br>*ρ* = -0.068<br>95%-CI [-0.292, 0.163]<br>*BF* = 0.309
H5b	Shapiro-Wilk test:<br>Variable 1:<br>*W* = 0.872<br>*p* = 0.013<br>Variable 2:<br>*W* = 0.88963<br>*p* = 0.026	Spearman correlation	1	*p* = 0.015<br>*ρ* =-0.550<br>95%-CI [-.803, -0.127]<br>*BF* = 4.84	*p* = 0.013<br>*ρ* = -0.546<br>95%-CI [-0.796, -0.137]<br>*BF* = 5.409
H6a (primary caregiver)	Shapiro-Wilk test:	Spearman correlation	4	*p* < .001<br>*ρ* =0.388	*p* = 0.003<br>*ρ* = 0.338

	Variable 1:<br>W = 0.97574<br>p = 0.1652<br>Variable 2:<br>W = 0.93584<br>p < 0.001			95%-CI [0.169, 0.571]<br>BF = 45.357	95%-CI [0.119, 0.526]<br>BF = 15.126
H6a (secondary caregiver)	Shapiro-Wilk test:<br>Variable 1:<br>W = 0.97574<br>p = 0.1652<br>Variable 2:<br>W = 0.93607<br>p = 0.003	Spearman correlation	4	p = 0.002<br>ρ =.398<br>95%-CI [0.158, 0.594]<br>BF = 6.18	p = 0.01<br>ρ = 0.323<br>95%-CI [0.082, 0.528]<br>BF = 2.674
H6b (primary caregiver)	Shapiro-Wilk test:<br>Variable 1:<br>W = 0.8478<br>p < 0.001<br>Variable 2:<br>W = 0.93584<br>p < 0.001	Spearman correlation	/	/	p = 0.132<br>ρ = 0.177<br>95%-CI [-0.054, 0.389]<br>BF = 0.762
H6b (secondary caregiver)	Shapiro-Wilk test:<br>Variable 1:<br>W = 0.8478<br>p < 0.001<br>Variable 2:<br>W = 0.93607<br>p = 0.003	Spearman correlation	/	/	p = 0.103<br>ρ = 0.207<br>95%-CI [-0.042, 0.207]<br>BF = 7.330
H6c (primary caregiver)	Shapiro-Wilk test:<br>Variable 1:<br>W = 0.915<br>p < 0.001<br>Variable 2:<br>W = 0.80232<br>p < 0.001	Spearman correlation	2	p = 0.002<br>ρ =0.354<br>95%-CI [0.133, 0.541]<br>BF = 20.345	p < 0.001<br>ρ = 0.401<br>95%-CI [0.190,0.577]<br>BF = 91.232
H6c (secondary caregiver)	Shapiro-Wilk test:<br>Variable 1:<br>W = 0.915<br>p < 0.001<br>Variable 2:<br>W = 0.8762<br>p < 0.001	Spearman correlation	/	/	p = 0.015<br>ρ = 0.3<br>95%-CI [0.062, 0.505]<br>BF = 0.911
H6d (primary caregiver)	Shapiro-Wilk test:<br>Variable 1:<br>W = 0.968<br>p = 0.064<br>Variable 2:	Spearman correlation	7	p = 0.114<br>ρ =-0.193<br>95%-CI [-0.413, 0.047]<br>BF = 0.877	p = 0.009<br>ρ = -0.300<br>95%-CI [-0.495, -0.077]<br>BF = 6.194

	W = 0.80232<br>*p* < 0.001				
H6d (secondary caregiver)	Shapiro-Wilk test:<br>Variable 1:<br>*W* = 0.968<br>*p* = 0.064<br>Variable 2:<br>*W* = 0.8762<br>*p* < 0.001	Spearman correlation	5	*p* = 0.866<br>*ρ* =-0.022<br>95%-CI [-0.268, 0.227]<br>*BF* = 0.303	*p* = 0.9<br>*ρ* = 0.016<br>95%-CI [-0.227, 0.257]<br>*BF* = 0.503
H7a	/	Spearman correlation was used because of ordinal scale	/	/	*p* = 0.363<br>*ρ* = 0.107<br>95%-CI [-0.124,0.328]<br>*BF* = 0.390
H7b	/	Spearman correlation was used because of ordinal scale	/	/	*p* = 0.268<br>*ρ* = -0.131<br>95%-CI [-0.349, 0.101]<br>*BF* = 0.470
H7c	/	Spearman correlation was used because of ordinal scale	6	*p* = 0.057<br>*ρ* =0.238<br>95%-CI [-0.007, 0.455]<br>*BF* = 1.505	*p* = 0.091<br>*ρ* = 0.202<br>95%-CI [-0.033, 0.416]<br>*BF* = 1.017
H8a (primary caregiver)	/	Spearman correlation was used because of ordinal scale	2	*p* = 0.104<br>*ρ* =0.193<br>95%-CI [-0.040, 0.407]<br>*BF* = 0.92	*p* = 0.086<br>*ρ* = 0.201<br>95%-CI [-0.029, 0.411]<br>*BF* = 1.047
H8a (secondary caregiver)	/	Spearman correlation was used because of ordinal scale	/	/	*p* = 0.427<br>*ρ* = -0.099<br>95%-CI [-0.333, 0.146]<br>*BF* = 0.382
H8b (primary caregiver)	/	Spearman correlation was used because of ordinal scale	/	/	*p* = 0.143<br>*ρ* = -0.172<br>95%-CI [-0.385, 0.059]<br>*BF* = 0.722
H8b (secondary caregiver)	/	Spearman correlation was used because of ordinal scale	/	/	*p* = 0.477<br>*ρ* = -0.091<br>95%-CI [-0.332, 0.16]<br>*BF* = 0.308

Exploratory 1 (year of birth & positive phrases heard)	Shapiro-Wilk test: Variable 1: $W = 0.88766$ $p < 0.001$ Variable 2: $W = 0.91198$ $p < 0.001$	Spearman correlation	1	$p = < .001$ $\rho = 0.390$ 95%-CI [0.176, 0.569] $BF = 60.56$	$p < .001$ $\rho = 0.413$ 95%-CI [0.204, 0.586] $BF = 133.89$
Exploratory 2 (year of birth & positive phrases said)	Shapiro-Wilk test: Variable 1: $W = 0.88766$ $p < 0.001$ Variable 2: $W = 0.943$ $p = 0.269$	Spearman correlation	1	$p = 0.352$ $\rho = -0.226$ 95%-CI [-0.617, 0.254] $BF = 0.680$	$p = 0.241$ $\rho = -0.275$ 95%-CI [-0.660,0.157] $BF = 0.817$
Exploratory 3 (low control phrases & warmth, primary caregiver)	Shapiro-Wilk test: Variable 1: $W = 0.8478$ $p < 0.001$ Variable 2: $W = 0.80232$ $p < 0.001$	Spearman correlation	2	$p = 0.007$ $\rho = -.317$ 95%-CI [-0.32, -0.09] $BF = 8.14$	$p = 0.034$ $\rho = -0.246$ 95%-CI [-0.25, -0.02] $BF = 0.762$
Exploratory 3 (low control phrases & warmth, secondary caregiver)	Shapiro-Wilk test: Variable 1: $W = 0.8478$ $p < 0.001$ Variable 2: $W = 0.8746$ $p < 0.001$	Spearman correlation	/	/	$p = 0.376$ $\rho = -0.111$ 95%-CI [-0.34, 0.14] $BF = 0.27$
Exploratory 4 (low warmth phrases & control, primary caregiver)	Shapiro-Wilk test: Variable 1: $W = 0.968$ $p = 0.064$ Variable 2: $W = 0.93584$ $p < 0.001$	Spearman correlation	5	$p = 0.023$ $\rho = 0.27$ 95%-CI [0.04, 0.48] $BF = 3.04$	$p = 0.004$ $\rho = 0.327$ 95%-CI [0.11, 0.52] $BF = 11.41$
Exploratory 4 (low warmth phrases & control, secondary caregiver)	Shapiro-Wilk test: Variable 1: $W = 0.968$ $p = 0.064$ Variable 2: $W = 0.94739$ $p = 0.007$	Spearman correlation	5	$p = 0.01$ $\rho = 0.32$ 95%-CI [0.08, 0.32] $BF = 5.09$	$p = 0.014$ $\rho = 0.32$ 95%-CI [0.07, 0.53] $BF = 3.94$
