# Supplementary material for: “Because I said so.” – Collection and evaluation of parenting phrases in German-speaking samples
Source: PLoS One. 2026 Apr 16;21(4):e0346718. doi: 10.1371/journal.pone.0346718 (PMC13086427; doi:10.1371/journal.pone.0346718)
Supplement: S2 Analysis — (PDF) [file pone.0346718.s007.pdf]

Prerequisite analyses Study 2 and results with and without outliers

Hypothesis	Prerequisite analysis	Statistical test used	Number of outliers	Results with outliers excluded	Results all data included
H1a	/	Spearman correlation was used because of ordinal scale	1	*ρ* =0.178<br>95%-CI [-0.014, 0.357]<br>*p* = 0.070<br>*BF* = 1.075	*ρ* = 0.19<br>95%-CI [0.00, 0.37]<br>*p* = 0.045<br>*BF* = 1.497
H1b	/	Spearman correlation was used because of ordinal scale	3	*ρ* = 0.066<br>95%-CI [-0.13, 0.26]<br>*p* = 0.509<br>*BF* = 0.28	*ρ* = 0.087<br>95%-CI [-0.11, 0.27]<br>*p* = 0.374<br>*BF* = 0.326
H1c	/	Spearman correlation was used because of ordinal scale	1	*ρ* =0.329<br>95%-CI [0.147 0.49]<br>*p* < .001<br>*BF* = 59.457	*ρ* = 0.329<br>95%-CI [0.15, 0.49]<br>*p* < .001<br>*BF* = 61.792
H2a	/	Spearman correlation was used because of ordinal scale	1	*ρ* = 0.245<br>95%-CI [0.037, 0.402]<br>*p* = 0.012<br>*BF* = 4.629	*ρ* = 0.240<br>95%-CI [0.05, 0.41]<br>*p* = 0.013<br>*BF* = 4.171
H2b	/	Spearman correlation was used because of ordinal scale	3	*ρ* = -0.172<br>95%-CI [-0.355, 0.023]<br>*p* = 0.084<br>*BF* = 0.939	*ρ* = -0.197<br>95%-CI [-0.37, -0.01]<br>*p* = 0.043<br>*BF* = 1.559
H2c	/	Spearman correlation was used because of ordinal scale	1	*ρ* = 0.109<br>95%-CI [-0.084, 0.294]<br>*p* = 0.268<br>*BF* = 0.402	*ρ* = 0.131<br>95%-CI [-0.06, 0.31]<br>*p* = 0.18<br>*BF* = 0.522
H3a	/	Spearman correlation was used because of ordinal scale	1	*ρ* = 0.002<br>95%-CI [-0.19, 0.19]<br>*p* = 0.983<br>*BF* = 0.2246	*ρ* = -0.021<br>95%-CI [-0.21, 0.17]<br>*p* = 0.832<br>*BF* = 0.228
H3b	/	Spearman correlation was used because of ordinal scale	2	*ρ* = -0.044<br>95%-CI [-0.24, 0.15]<br>*p* = 0.654<br>*BF* = 0.248	*ρ* = -0.027<br>95%-CI [-0.22, 0.16]<br>*p* = 0.783<br>*BF* = 0.232
H3c	/	Spearman correlation was used because of ordinal scale	2	*ρ* = -0.036<br>95%-CI [-0.23, 0.16]<br>*p* = 0.715<br>*BF* = 0.24	*ρ* = -0.019<br>95%-CI [-0.21, 0.17]<br>*p* = 0.85<br>*BF* = 0.228
H4a	Levene's test:	Independent t-test	1	*t*(103) = 0.345<br>*p* = 0.731	*t*(104) = 0.54<br>*p* = 0.591<br>95%-CI [-0.08, 0.14]

	F(1, 104) = 0.537<br>*p* = 0.465<br>(robustness to non-normality due to sample size)			95%-CI [-0.09, 0.13]<br>*BF* = 0.222<br>*d* = 0.07	*BF* = 0.239<br>*d* = 0.11
H4b	Levene's test:<br>*F*(1, 104) = 5.299<br>*p* = 0.023<br>(robustness to non-normality due to sample size)	Welch's test	3	*t*(99.505) = -1.39<br>*p* = 0.166<br>95%-CI [-0.09, 0.13]<br>*BF* = 0.454<br>*d* = -0.26	*t*(102.69) = -1.56<br>*p* = 0.122<br>95%-CI [-0.27, 0.03]<br>*BF* = 0.541<br>*d* = -0.30
H4c	Levene's test<br>*F*(1, 104) = 0.58<br>*p* = 0.45<br>(robustness to non-normality due to sample size)	Independent t-test	1	*t*(103) = -0.33<br>*p* = 0.74<br>95%-CI [-0.12, 0.08]<br>*BF* = 0.22<br>*d* = - 0.07	*t*(104) = 0.04<br>*p* = 0.97<br>95%-CI [-0.11, 0.11]<br>*BF* = 0.210<br>*d* = 0.01
Exploratory 1a (gender differences warmth rating)	Shapiro-Wilk test:<br>Group 1:<br>*W* = 0.9.82<br>*p* = .933<br>Group 2:<br>*W* = 0.982<br>*p* = .290<br>Levene's test:<br>*F*(1, 104) = 1.5417<br>*p* = 0.217	Independent t-test	1	*t*(103) = 2.31<br>*p* = 0.011<br>95%-CI [0.04, inf]<br>*M*(men) = 2.89<br>*M*(women) = 2.75<br>*BF* = 2.346<br>*d* = 0.54	*t*(104) = 2.10<br>*p* = 0.019<br>95%-CI [0.03, inf]<br>*M*(men) = 2.89<br>*M*(women) = 2.76<br>*BF* = 1. 564<br>*d* = 0.49
Exploratory 1b (gender differences control rating)	Shapiro-Wilk test:<br>Group 1:<br>*W* = 0.907<br>*p* = .030<br>Group 2:<br>*W* = 0.958<br>*p* = .009<br>Levene's test:	Mann-Whitney U test	3	*W* = 947<br>95% CI [-0.119, inf]<br>*p* = 0.380<br>*BF* = 0.254	*W* = 1027<br>95% CI [-0.119, inf]<br>*p* = 0.373<br>*BF* = 0.251

	F(1, 104) = 1.5417<br>*p* = 0.217				
Exploratory 1c (gender differences self-esteem-enhancement rating)	Shapiro-Wilk test:<br>Group 1:<br>*W* = 0.9.18<br>*p* = .053<br>Group 2:<br>*W* = 0.987<br>*p* = .572<br>Levene's test:<br>*F*(1, 104) = 0.30<br>*p* = 0.59	Independent t-test	1	*t*(103) = 3.00<br>*p* = 0.002<br>95%-CI [0.08, inf]<br>*M*(men) = 2.95<br>*M*(women) = 2.78<br>*BF* = 14.118<br>*d* = 0.73	*t*(104) = 3.65<br>*p* < 0.001<br>95%-CI [0.12, inf]<br>*M*(men) = 3.00<br>*M*(women) = 2.78<br>*BF* = 63.191<br>*d* = 0.85
Exploratory 2a (phrases: warmth & control	/	Spearman correlation was used because of ordinal scale	/	/	*ρ* = 0.001<br>95 % CI [-0.19, 0.19]<br>*p* = 0.995<br>*BF* = 0.224
Exploratory 2b (phrases: warmth & self-esteem-enhancement)	/	Spearman correlation was used because of ordinal scale	/	/	*ρ* = 0.660<br>95 % CI [0.54, 0.76]<br>*p* < .001<br>*BF* = 373937601196
Exploratory 2c (phrases: control & self-esteem-enhancement)	/	Spearman correlation was used because of ordinal scale	/	/	*ρ* = -0.051<br>95 % CI [-0.24, 0.14]<br>*p* = 0.603<br>*BF* = 0.25
Exploratory 3a (dimensions: warmth & control)	/	Spearman correlation was used because of ordinal scale	/	/	*ρ* = -0.697<br>95%-CI [-0.79, -0.57]<br>*p* < .001<br>*BF* = 2.22
Exploratory 3b (dimensions: warmth & self-esteem-enhancement)	/	Spearman correlation was used because of ordinal scale	/	/	*ρ* = 0.972<br>95%-CI [0.96, 0.98]<br>*p* < .001<br>*BF* = 5.046
Exploratory 3c (dimensions: control & self-esteem-enhancement)	/	Spearman correlation was used because of ordinal scale	/	/	*r* = -0.687<br>95%-CI [-0.79, -0.55]<br>*p* < .001<br>*BF* = 9681608909
